# Effects of Wearing Personal Protective Equipment on Serum Cortisol Levels and Physiological Variables in Healthcare Workers: A Randomised Controlled Trial in a Simulated Pandemic Environment

**DOI:** 10.7759/cureus.61687

**Published:** 2024-06-04

**Authors:** Pradip Barde, Vinay Chitturi, Gaurav Sharma, Naresh Parmar, Rajesh Kathrotia, Deepak Parchwani, Vivek K Sharma

**Affiliations:** 1 Physiology, All India Institute of Medical Sciences Rajkot, Rajkot, IND; 2 Physiology, All India Institute of Medical Sciences, Rajkot, Rajkot, IND; 3 Biochemistry, All India Institute of Medical Sciences, Rajkot, Rajkot, IND

**Keywords:** simulated environment, covid-19, health care workers, hrv, cortisol, stress, full body gown type ppe

## Abstract

Introduction: The COVID-19 pandemic has necessitated the widespread use of personal protective equipment (PPE), particularly in high-risk environments. Full-body PPE is favoured for its comprehensive protection against the virus but poses challenges to the body's thermoregulatory system as it inhibits air exchange. This randomised trial was undertaken to investigate the effects of wearing a commonly used gown-type full-body PPE kit in a simulated environment.

Methods: Initially, 65 healthy males were recruited and randomly divided into two groups: a study group wearing a full-body PPE kit (gown-type, full-body PPE kit with trousers, a gown-type shirt with a hood, a shoe cover, an N95 face mask, and an optional face shield) and a control group without PPE. They remained seated for three hours while wearing the PPE kit. Room conditions mimicked non-air-conditioned hospital scenarios, with temperature and humidity recorded and ventilation provided through open doors and windows, along with ceiling fan cooling. Activities with minimal physical exertion were allowed, and access to the toilet was kept to a minimum. Subjects underwent assessments of heart rate, respiratory rate, temperature, blood pressure, heart rate variability (HRV), and blood samples for serum cortisol before donning the PPE kit and entering a simulated ICU/WARD environment and after doffing.

Results: A total of 60 participants completed the study (30 in each group). Compared to the controls, serum cortisol levels significantly increased in the PPE groups, and HRV data indicated increased sympathetic activity in the PPE group.

Conclusion: Wearing a full-body PPE kit (gown-type upper garment with trousers) was found to have a significant impact on cortisol levels and physiological variables in a simulated environment. This suggests that in situations like the COVID-19 pandemic that warrant the use of such PPE kits, appropriate measures should be taken to provide better thermal stability for maintaining the well-being of healthcare workers.

## Introduction

The COVID-19 pandemic and similar situations require the use of personal protective equipment (PPE) kits. Among the variety of PPE kits available, full-body kits are preferred in these situations because of their increased level of protection. However, the impervious nature of the fabric and design of these kits can cause thermal stress, which can affect human performance due to its impact on various body parameters [1,2]. A study, which reviewed the literature on thermal stress in healthcare workers wearing full-body PPE kits during the COVID-19 pandemic, found that thermal stress is a common problem among healthcare workers wearing PPE kits, and that it can lead to a variety of health problems, including dehydration, heat exhaustion, and heat stroke [2]. A recent study found that wearing a full-body PPE kit led to significant increases in heart rate, respiratory rate, and body temperature, as well as decreases in oxygen saturation and cognitive performance [3]. Another study found that the psychological and biochemical effects of wearing a full-body PPE kit were significant, with participants reporting increased levels of stress, anxiety, and fatigue [4]. A systematic review by Radha K et al. on the physiological and psychological impacts of wearing full-body PPE kits on healthcare workers found that wearing PPE kits can lead to a variety of negative effects, including increased heart rate, respiratory rate, and body temperature; decreased oxygen saturation; and increased levels of stress, anxiety, and fatigue [5]. A review also found that comorbidities like obesity, hypertension and diabetes can increase the adverse effects of PPE kits and interfere with cognitive function and work performance [6].

Unlike single-unit full-body PPE kits, the gown-type upper garment PPE kits with separate lower trousers are a good option for use in low-risk and moderate-risk situations. They are comfortable, breathable, and cost-effective, though they do not provide as high a level of protection as single-unit full-body PPE kits. They may be associated with less thermal stress. However, there is a lack of studies regarding the impact of wearing the most commonly used gown-type upper garment PPE kit on various parameters. Hence, this randomised trial was planned to study the impact of wearing a full-body gown-type PPE kit on physiological and biochemical variables in a simulated pandemic environment.

## Materials and methods

Study design and settings

This study is a randomized control trial conducted in the Department of Physiology AIIMS Rajkot in collaboration with the Department of Microbiology, PDU Medical College and Hospital in Rajkot, India. The study was approved by the Institutional Ethics Committee (O. W. NO./AIIMS/ RAIKOT/ IEC/10/2021 dated 15/09/2021). The trial was registered prospectively with the Clinical Trial Registry of India (CTRI/2021/09/036833, registered on 24/09/2021).

Recruitment of participants

Study Population

The initial study population comprised 70 apparently healthy male healthcare workers in the age group of 18-55 years with no history of any acute or chronic illness and without any addictions were considered for the study. A consecutive sampling method was used. The participants were requested to visit the Department of Physiology at their convenience. After initial screening as per inclusion criteria a total of 65 healthy male participants were recruited and informed written consent was obtained.

Randomization and Allocation

**Figure 1:**
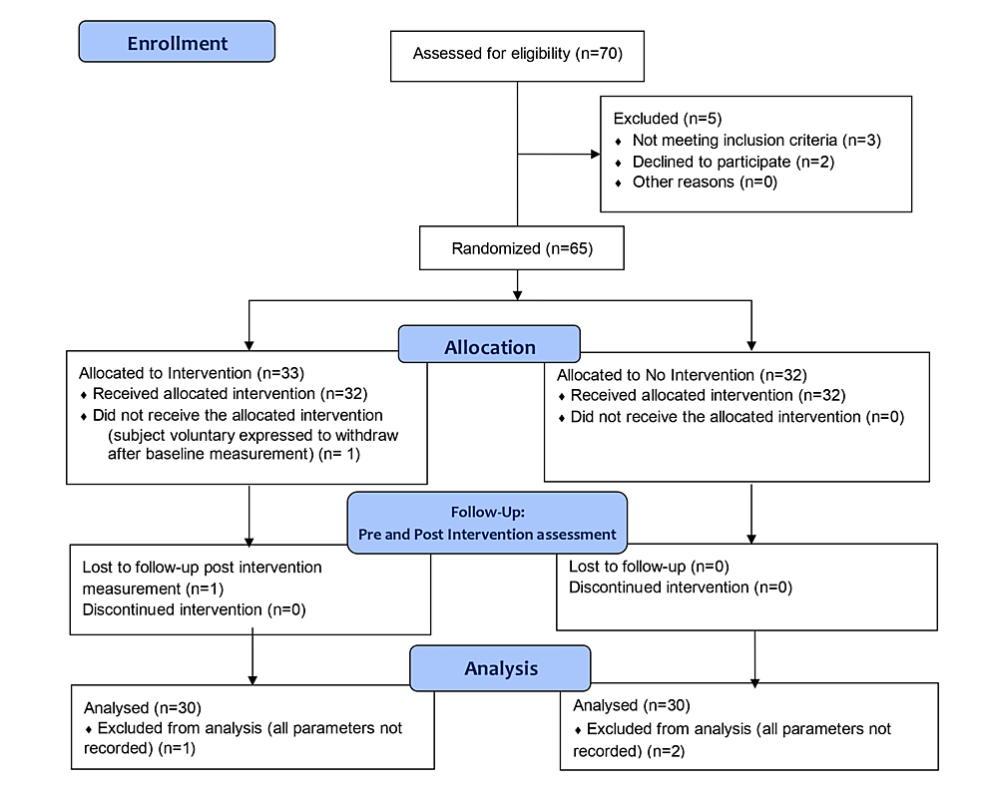
Study Flow Chart

The study was planned with two groups, a study group who would wear a full-body PPE kit (gown-type, full-body PPE kit with trousers, a gown-type shirt with a hood, a shoe cover, an N95 face mask, and an optional face shield) and a control group without a PPE kit. The participants were randomly divided into a PPE group and a non-PPE control group using the block randomisation method. Thirty-three participants were assigned to the study group and 32 to the control group. The flow chart for the study is shown in Figure 1. The participants who completed the study protocol were included in the final analysis as per protocol basis.

Parameters Measured

A set of instructions was given to the study subjects which included maintaining a normal work routine, having regular meals at specific times, avoiding tea, coffee, and alcoholic beverages, and ensuring seven to eight hours of sleep the night before the test day.

Baseline Measurements

On the test day, subjects were asked to report between 7 am and 8 am. This was followed by baseline recording. After this, they were allowed to rest for 10 min, in a sitting position, after which 5 min ECG recording was done for HRV measurement (Model DYN71; Dinamika Technologies, St. Petersburg, Russia). Later, their blood pressure was measured using an automated non-invasive blood pressure (NIBP) monitor as part of a multiparameter monitor (Model TruSkan S500, Sr. No. H22GH5194 and H22GH5191, Skanray Healthcare Pvt Ltd., Mysuru, India), along with monitoring of electrocardiography (ECG), pulse oximetry (SPO2), and end-tidal carbon dioxide measurement (ETCO2). The body temperature was measured with a non-contact tympanic membrane thermometer (Genius 3, Cardinal Health, Inc., Dublin, Ireland). The values of the physiological variables were recorded at baseline, and then at intervals of one hour each for three hours and after complete doffing.

For biochemical parameter (serum cortisol) estimation, 5 ml of venous blood was withdrawn from the anterior cubital vein at baseline and after doffing in vacuum tubes without anticoagulant to separate serum. The separated serum after centrifugation, was labelled and stored at -80ºC. Cortisol levels were done on stored serum in the Department of Microbiology. The enzyme-linked immunosorbent assay (ELISA) method was used for estimation (ELISA kit supplied and manufactured by Xema, Shinya, Beijing, China; Aichwald, Germany).

Intervention

For the study group, the standard, full-body PPE kits (gown-type, full-body PPE kit with trousers, a gown-type shirt with a hood, a shoe cover, an N95 face mask, and an optional face shield) were used as per the Unique Certification Code (UCC-COVID19) of PPE garments and fabric which passed the laboratory tests laid down by the South India Textile Research Association (SITRA) as well as the Defence Research and Development Establishment (DRDE). The most commonly available and used gown-type PPE kit included a full-body PPE kit with trousers, a gown-type shirt with a hood, a shoe cover, an N95 face mask, and an optional face shield. As per the hospital scenario in COVID-19 hospital wards, there was no air-conditioning in our study setup. The room temperature and humidity were recorded throughout the study with ventilation through open doors and windows along with cooling by ceiling fans.

During the study, the subjects were asked to sit on a chair and not to do any activities involving more than minimal physical exertion like the use of a cell phone for listening to music, reading books, or reading a newspaper. To mimic real-life duty situations, access to the washroom was restricted to emergency cases. Drinking water was allowed once to mimic real-life situations. No eating or drinking of any other beverage was allowed.

After the completion of three hours and recording of physiological measurements, the doffing was done and the blood samples were collected immediately. Then, after a 10-minute rest, the final reading for physiological measurements was taken.

The control group participants also reported at the same time of the day as the study participants and underwent a similar recording process for all parameters and blood sample collection without wearing the PPE kit. 

Out of 33 participants in the study group, one participant withdrew from the study after baseline measurement. One participant refused to undergo post-intervention measurements while one participant was excluded as all the parameters were not recorded. In the control group, two participants were excluded as all parameters were not recorded.

Statistical analysis

The data was tabulated in Microsoft Excel. To perform the statistical analysis, Jamovi (Version 2.2.2, The Jamovi Project, Sydney, Australia) and Excel (Version 365, Microsoft, Redmond, USA) were used. Data was checked for normal distribution with the Shapiro-Wilk test. As data was not normally distributed, non-parametric tests were used for analysis. Statistical comparison within groups was performed using the Wilcoxon signed ranked test and between groups using the Mann-Whitney U test. A difference was estimated to be statistically significant at p < 0.05.

## Results

A total of 60 males with a mean age of 28.19±6.40 years (range 21-48 years) completed the study protocol. On anthropometric assessments, participants had an average body mass index (BMI) of 24.18±3.51 kg/m2 and the average body percentage of body fat was 20%.

**Figure 2:**
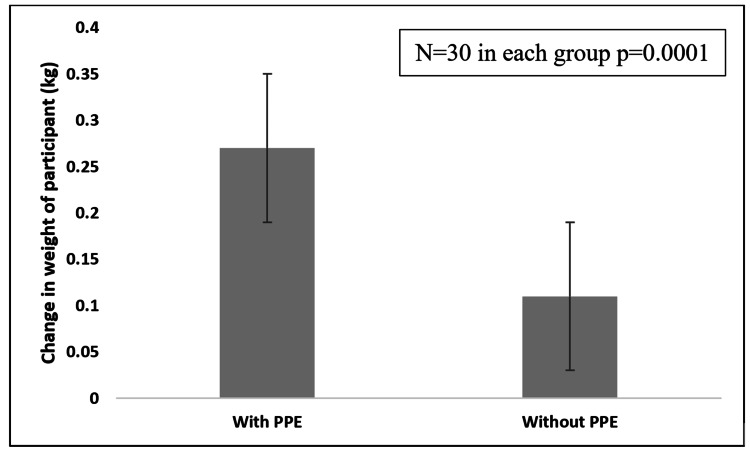
Comparison of change in weight of participants with and without PPE PPE: personal protective equipment

Figure 2 shows a significant change in the weight of participants from baseline before donning the PPE to that after doffing the PPE as compared to the control group.

**Figure 3:**
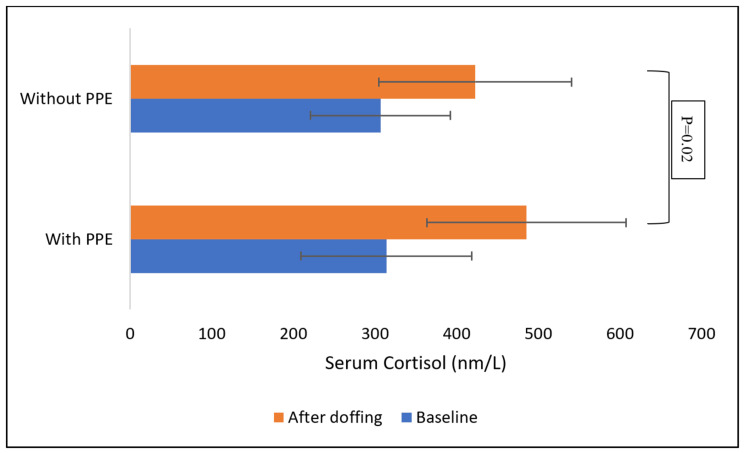
Comparison of change in the serum cortisol of participants with and without PPE. PPE: personal protective equipment

The change in serum cortisol levels in the PPE group and control group is shown in Figure 3. Serum cortisol level in the control group and PPE group at baseline was 313.8 nm/l and 308nm/l respectively and post-exposure serum cortisol in the PPE group was 485.2 nm/l and in the control group was 422.9 nm/l. The change in serum cortisol after the intervention was statistically significant for both the groups compared to baseline (p=0.0001) and serum cortisol was significantly increased after exposure in the PPE group compared to the control group (p=0.02).

The skin temperature and core temperature of the participants along with room temperature and humidity expressed as mean and standard deviation are given in Table 1, in the PPE group and control group. The table shows that the average temperature of the skin is 36.3 degrees Celsius, and the average temperature of the room is 30.9 degrees Celsius in the PPE group. The mean room humidity was 77.2% with PPE and 65.9% without PPE.

**Table 1:** Mean temperature of the study participants (values are mean±SD). PPE: personal protective equipment

Temperature Parameters (degree Celsius)	PPE group (N=30)	Control group(N=30)
Core temperature	36.7±0.1	36.6±0.1
Skin temperature	36.3±0.1	36.3±0.0
Room temperature	30.9±0.2	31.3±0.2

Table 2 shows the physiological paraments such as heart rate (HR), systolic blood pressure (SBP), diastolic blood pressure (DBP), respiratory rate (RR), end-tidal carbon dioxide (ETCO2), and oxygen saturation (SPO2) of participants with and without personal protective equipment (PPE). The measurements were taken at baseline, immediately after exercise, and after rest. The heart rate (HR) and systolic and diastolic blood pressures (SBP and DBP) showed no significant differences between the two conditions, except for a marginal difference in SBP immediately after the activity. Respiratory rate (RR) exhibited a marginal increase in individuals wearing PPE immediately after the study. End-tidal CO2 (ETCO2) levels were similar in both groups during the intervention and not statistically significant. However, ETCO2 was significantly lower in the PPE group after rest. Oxygen saturation (SPO2) showed significant differences in all conditions, with lower values in the PPE group. Skin, Core, and Room temperature were not much different among the two groups. Humidity demonstrated significant variations, with the PPE group experiencing higher values in both parameters. These findings highlight the potential impact of PPE on physiological parameters and underscore the importance of considering environmental and protective factors in healthcare settings.

**Table 2:** Comparison of physiological variables in study participants (values are mean±SD). PPE: Personal protective equipment, HR: heart rate, SBP: systolic blood pressure, DBP: diastolic blood pressure, RR: respiratory rate, EtCO2: end-tidal carbon dioxide, SpO2: oxygen saturation

Physiological Parameters		PPE group (N=30)	Control group (N=30)	P-value	
		Mean±SD	Mean±SD		
HR (beats/min)	Baseline	85.06±2.25	89.67±2.16	0.06	
	Immediately after	84.39±2.06	85.00±2.06	0.57	
	After rest	83.74±1.92	84.60±2.09	0.46	
SBP (mmHg)	Baseline	121.77±1.85	118.47±4.19	0.41	
	Immediately after	122.16±1.73	119.27±1.75	0.09	
	After rest	118.52±1.44	120.77±1.90	0.31	
DBP (mmHg)	Baseline	78.77±1.46	78.40±1.61	0.76	
	Immediately after	78.32±1.34	76.17±1.55	0.08	
	After rest	78.77±1.54	77.97±1.17	0.46	
Respiratory rate per min	Baseline	17.84±0.52	20.00±0.72	0.07	
	Immediately after	19.61±0.69	19.80±0.79	0.88	
	After rest	18.58±0.65	18.47±0.67	0.86	
Core temperature (degree Celsius)	Baseline	36.72±0.06	36.52±0.06	0.12	
	Immediately after	36.68±0.07	36.61±0.07	0.27	
	After rest	36.68±0.06	36.67±0.08	0.90	
ETCO2 (mmHg)	Baseline	36.26±0.51	37.23±0.63	0.13	
	Immediately after	36.16±0.65	37.40±0.63	0.08	
	After rest	35.77±0.55	37.27±0.50	0.01	
SPO2 (%)	Baseline	98.00±0.00	98.17±0.16	0.31	
	Immediately after	98.06±0.22	98.27±0.22	0.01	
	After rest	97.74±0.38	98.07±0.21	0.01	
Room temperature (degree Celsius)	Baseline	30.49±0.32	31.23±0.21	0.11	
	Immediately after	31.11±0.36	31.36±0.18	0.54	
	After rest	31.11±0.36	31.30±0.15	0.64	
Humidity	Baseline	77.84±1.42	66.60±1.29	<0.01	
	Immediately after	76.93±1.64	65.50±1.17	<0.01	
	After rest	76.71±1.57	65.47±1.12	<0.01	
Skin temperature (degree Celsius)	Baseline	36.33±0.02	36.31±0.02	0.34	
	Immediately after	36.37±0.02	36.34±0.02	0.06	
	After rest	36.32±0.02	36.31±0.02	0.89	

Table 3 presents an analysis of heart rate variability (HRV) parameters for individuals in the PPE and control group, emphasizing both intra-group and inter-group comparisons. The table includes medians and interquartile ranges (IQR) for various HRV metrics, such as heart rate (HR), standard deviation of NN intervals (SDNN), root mean square of successive differences (RMSSD), NN50, pNN50 (%), high-frequency power (HF), low-frequency power (LF), normalized HF power (HFnu), normalized LF power (LFnu), LF/HF ratio, total power (TP), and HRV index. Intra-group comparisons (before and after in the PPE group; P-value<0.05) reveal statistically significant changes in multiple parameters including SDNN, RMSSD, NN50, pNN50, HF, LF and HRV index. The inter-group analysis highlights significant differences in HFnu, and LF/HF ratio (PPE group vs control group; P-value<0.05).

**Table 3:** Comparison of HRV parameters in the PPE group and control group (before donning and after doffing of PPE) *P-value on comparison of parameters before donning and after doffing. ^P<0.05 on comparison of the control and the PPE groups. PPE: personal protective equipment; IQR: interquartile range; HRV: heart rate variability; HR: heart rate; SDNN: standard deviation of NN intervals; HF: high frequency; LF: low frequency; TP: total power; LFnu: LF normalized units, HFnu: high frequency normalized units NN50 refers to the change in successive normal sinus (NN) intervals exceeding 50 ms. pNN50 refers to the proportion of NN50 divided by the total number of NN (R-R) intervals. RMSSD refers to the root mean square of successive differences between normal heartbeats.

HRV parameters	PPE group (N=30)		*P- value	Control group (N=30)		*P-value
	Before donning	After doffing		Before donning	After doffing	
	Median (IQR)	Median (IQR)		Median (IQR)	Median (IQR)	
HR (beats/min)	84.00 (11.75)	81.00 (17.25)	0.09	88.00 (10.75)	78.50 (16)	0.66
SDNN (ms)	38.70 (18.37)	41.50 (20.47)	0.03	28.75 (16.52)	44.10 (20.32)	0.36
RMSSD (ms)	23.7 (17.15)	24.3 (21.1)	0.01	16.3 (10.88)	23.5 (14.78)	0.62
NN50	13.50 (32.75)	16.50 (44.25)	0.02	3.00 (12)	13.50 (25.75)	0.43
pNN50 (%)	4.50 (11)	6.00 (14.75)	0.02	1.00 (4)	4.50 (8.75)	0.48
HF (ms²)	202.50 (252.5)	212.00 (343.5)	0.02	93 (100.75)	163 (297)	0.29
LF (ms²)	617.50 (604)	764.00 (871)	0.05	320 (458)	780 (636)	0.18
HFnu	19.51 (17.61)	23.16 (13.93)	0.80	22.27 (17.53)	18.19 (17.55) ^	0.04
LFnu	80.50 (17.61)	76.85 (13.93)	0.80	77.74 (17.96)	81.81 (18.14)	0.17
LF/HF	4.13 (4.62)	3.35 (3.21)	0.85	3.72 (4.47)	4.65 (5.96) ^	0.02
TP (ms²)	1175 (1652)	1366 (1798)	0.11	794.5 (838.5)	1663 (1679.75)	0.26
HRV index	11.00 (4)	9.50 (4.75)	0.01	7.00 (4)	10.50 (3.75)	0.10

## Discussion

The present study offers valuable insights into the physiological impact of full-body gown-type personal protective equipment (PPE) on human responses. This study demonstrated a significant change in serum cortisol levels, respiratory rate and SPO2 with PPE compared to without PPE.

Thermal stress

The observed weight loss in the PPE group, as compared to the control group's stable weight, indicates that there was water loss from the body in the form of sweating and insensible water loss from the body as part of thermoregulation in order to maintain normal body temperature. These findings align with existing literature. A cross-sectional study on the use of PPE kits has recorded that excessive sweating was a very common discomfort associated with PPE [4] The evaporation of sweat is the most critical heat transport mechanism which gets severely affected by protective clothing which are evaporation resistant [2,7]. The rate of metabolic heat production, sweating and evaporation of sweat is affected by clothing design, clothing fit and clothing air permeability [8,9]. The evaporation-resistant material cloth of full-body PPE kits leads to excessive sweating. This suggests heightened thermoregulatory demands due to PPE-induced heat-trapping and subsequent sweating to maintain body temperature [10,11].

Similar to our study, researchers have shown that while wearing PPE the median participants' body temperature was 0.1°C higher compared to not wearing PPE and this difference varied with change in ambient temperature [12]. The data encompassing the temperature of skin, core, room, and humidity, effectively captures the thermal context of PPE usage [2,7]. As expected, the higher skin temperature in the PPE group reflects the insulating properties of the gear [11]. The additional room temperature and humidity data provide essential context for interpreting the thermal environment contributing to thermal stress with PPE [7,13].

Serum cortisol

While both groups demonstrated significantly increased cortisol levels after the study, there was a significant difference in the increase in serum cortisol levels in the PPE group as compared with the non-PPE group. Cortisol, a stress hormone, typically increases during stressful conditions and has deleterious effects on mental and physical health. Further research is needed to isolate the contribution of PPE use from potential stressors associated with the study itself [14-16]. This is crucial given the potential impact of non-PPE stressors on cortisol elevation.

Physiological variables

While heart rate and blood pressure remained unchanged, the marginal increase in respiratory rate (RR) after PPE use may be a mechanism for heat loss and heat-induced stress, it warrants further investigation [13]. A minimal change in heart rate or even a linear relationship of heart rate with temperature with or without PPE has been documented [10,12]. A systematic review suggested increase in heart rate with PPE was highly dependent on the weight of the PPE, the type of cloth of PPE and weather conditions [17]. No change in cardiovascular and respiratory parameters was noted with the use of N95 masks in a study of pregnant vs non-pregnant females [18]. Thus, lightweight PPE used in our study and no difference in temperature with or without PPE might explain unchanged heart rate and blood pressure in the PPE group as well as the control group. 

The end-tidal CO2 (ETCO2) levels were not statistically significant in the PPE vs control group at baseline or immediately after intervention. In the literature we found a study that showed ETCO2 level was higher with PPE compared to without PPE, moreover, it declined after removal of the PPE [10]. Our study found no significant increase in ETCO2 with PPE but a significantly lower level was noted after the removal of PPE. Significantly lower ETCO2 in the PPE group after rest may be a result of an increase in CO2 washout by the ventilatory response to stress which requires further exploration. [19,20]. We found SPO2 to be decreased in the PPE group during and after intervention similar to previous studies. In a study of nurses who wore PPE with N95 and coverall hazmat, SPO2 was found to be decreased by 2% and was associated with significant complaints of breathing difficulty because it can influence air circulation and ventilation process and thus cause hypoxia [21]. In a similar study, SPO2 levels of 98.57% before wearing PPE decreased to 98.34% with PPE and after the removal of PPE it was 98.39% - without major variation, it remained around 98% [10]. Oxygen consumption have been shown to be increased with wearing a PPE kit [17]. Significantly lower SpO2 in the PPE group compared to the control group across conditions might explain the tiredness or fatigue with PPE use as documented [21], however, it is interesting that overall the levels are in the normal range in our study which might be explained by the less restrictive type of full-body PPE kit. However, this necessitates further research to elucidate the underlying mechanisms accounting for its decrease [19,21].

Heart Rate Variability

The elevated LF/HF ratio in the PPE group suggests a potential shift towards sympathetic dominance, aligning with studies linking altered autonomic balance to stress and increased sympathetic activity [22,23]. Medical staff working in shifts during the COVID-19 pandemic and wearing PPE kits showed a significant decline in time domain parameters of HRV like SDNN and RMSSD which is in contrast to our study [24]. This decline in the HRV parameters might be explained by the severe stressful work routine during COVID-19 situation compared to our simulation where participants were not involved in active work. Overall, the increase in HRV values in the PPE group after PPE use is intriguing as it indicates the complex nature of the autonomic response to PPE and to resting idle during the study period [23]. Future studies could explore the specific stressors contributing to this shift and its potential implications for health and performance in real-life scenarios.

Limitations and future directions

The study has limitations in that the participants were kept inactive during the study period; working in a real-life environment would involve some activity. A future simulated environment may be planned keeping this in mind. Overall, the study provides valuable data on the physiological effects of PPE, but further research is needed to isolate the specific contribution of PPE from other potential stressors and delve deeper into the observed changes in respiratory rate, ETCO2, SpO2, and autonomic balance.

## Conclusions

This randomised controlled trial provides a comprehensive overview of the stress involved with wearing a PPE kit in a simulated environment. These findings of impact on weight, serum cortisol levels and various physiological parameters can be valuable contributions to the ongoing discourse on health implications of prolonged PPE use in various settings and can provide a base for further studies.
